# Changes in healthcare engagement during the COVID-19 pandemic

**DOI:** 10.1186/s41687-025-00850-z

**Published:** 2025-02-20

**Authors:** McKenzie Lockett, Gisselle C. Tamayo, Benjamin D. Schalet, Steven P. Reise, Rachel Kimerling

**Affiliations:** 1 National Center for PTSD, VA Palo Alto Health Care System, PTSD-334, 795 Willow Road, Menlo Park, CA 94025 USA; 2 Department of Psychiatry and Behavioral Sciences, Stanford University School of Medicine, Stanford, CA USA; 3 Center for Innovation to Implementation, VA Palto Alto Health Care System, Menlo Park, CA USA; 4 Department of Epidemiology and Data Science, Amsterdam UMC, Vrije Universiteit, Amsterdam, The Netherlands; 5 Department of Psychology, University of California Los Angeles, Los Angeles, CA USA

**Keywords:** Patient-centered care, VA healthcare system, Quality indicators

## Abstract

**Background:**

Healthcare engagement, defined as the self-efficacy to enact the behaviors needed to obtain optimal benefit from health services, is an important aspect of healthcare quality. Measuring changes to healthcare engagement is essential to informing current and ongoing adaptations to health service delivery. The objective of the present study was to explore the responsiveness of the recently developed PROMIS^®^ Healthcare Engagement measure (PHE), a patient-reported outcome, through investigating the impact of COVID and COVID-related healthcare disruptions on healthcare engagement from pre- to peri-pandemic.

**Methods:**

Baseline data (2018–2019) were collected via a national mail survey of Veterans receiving VA care. For follow-up data, a subset of participants was randomly selected to be invited to a follow-up survey. Administrative data was used from the VA’s Corporate Data Warehouse (CDW). We used mixed effects linear modeling to compare changes in healthcare engagement from baseline to follow-up between Veterans who reported healthcare disruptions and Veterans who did not report healthcare disruptions, adjusting for covariates.

**Results:**

From baseline to follow-up, healthcare engagement scores increased on average by 2.84 points. Compared to Veterans who reported no disruptions, Veterans who experienced COVID-related healthcare disruptions demonstrated greater decreases to healthcare engagement (difference scores ≥ − 1.98, *p*s ≤ 0.002) Further, Veterans with more healthcare disruptions showed greater decreases in healthcare engagement relative to those with fewer healthcare disruptions, such that Veterans with 2 healthcare disruptions (difference score = -4.20) significantly differed from Veterans reporting only 1 healthcare disruption, and Veterans reporting 3 or more disruptions (difference score = -3.75) significantly differed from those with 2 disruptions.

**Conclusion:**

Our results provide preliminary evidence of the PHE’s responsiveness through demonstrating that environmental factors, such as pandemic-related factors, influence healthcare engagement. The COVID-19 pandemic had a complex effect on healthcare engagement, with healthcare engagement scores increasing overall during the pandemic but Veterans reporting COVID-related healthcare disruptions showing decreased changes in healthcare engagement. These findings support the utility of the PHE as a measure of healthcare engagement.

**Supplementary Information:**

The online version contains supplementary material available at 10.1186/s41687-025-00850-z.

## Introduction

Healthcare engagement reflects the collaborative, bi-directional processes where care is delivered in partnership with patients and their families. Active engagement in one’s healthcare is essential to benefitting from health services, particularly for people with chronic conditions who have to navigate complex care plans, interactions with multiple clinics and specialists, and frequent healthcare encounters [12, 13, 17]. At the individual level, engagement can be considered as the self-efficacy to enact the health and healthcare behaviors required to receive optimal benefit from health services [17]. Rather than a static individual trait, patient healthcare engagement is reciprocally determined by the strengths and vulnerabilities of the patient and by the resources and usability characteristics of the healthcare systems, settings, and providers [12]. Consequently, healthcare engagement is a context-dependent construct and can be influenced by both changes to patient characteristics, such as changes to patient activation, as well as changes in the broader healthcare system.

The Patient-Reported Outcomes Measurement Information System (PROMIS^®^) Healthcare Engagement (PHE) measure is a recently developed measure of healthcare engagement [18]. The PHE assesses healthcare engagement behavior related to three interrelated domains: collaborative communication with providers, preventive and self-management behaviors, and navigation of health systems [18, 24]. Content validity for the PHE was derived through expert reviews and qualitative concept elicitation with a sample of Veterans with chronic and mental health condition [17]. Items were constructed at a sixth-grade reading level and cognitive interviewing was conducted to ensure reading comprehension [24]. Prior psychometric validation for the PHE item bank has confirmed its unidimensional factor structure, calibrated the measure using item response theory (IRT) and documented measurement invariance across age, race, gender, as well as for individuals receiving care for mental health vs. chronic conditions [24]. A subsequent study focused on the 8-item short form and prospective study of Veterans also demonstrated the established internal consistency, test-retest reliability, and construct validity. The PHE demonstrated good convergent validity with measures of provider communication, self-management and maintaining a usual source of care. The PHE also demonstrated excellent predictive validity over 1-year, where higher scores predicted better engagement behaviors such as lower primary care and mental health no-show rates, use of the patient portal, better medication adherence and better control of chronic conditions [18]. Though there is strong evidence for the reliability and validity of the PHE, the responsiveness of the measure has yet to be investigated.

Responsiveness refers to an instrument’s ability to accurately detect change over time in the construct of interest [22, 27]. This is particularly important if the PHE is to be used to evaluate the effectiveness of clinical interventions [19]. As healthcare systems increasingly focus on implementing strategies for engaging patients as active collaborators in their care [4, 5, 12], we must be able to evaluate the success of these efforts by whether they result in meaningful changes to healthcare engagement. One of the strengths of the PHE is the ability to evaluate engagement over a broad range of conditions and treatment, but such studies typically require large samples of healthcare users. Health services research places a high value on the generalizability from “real-world evidence” from well-controlled observational studies [3]. Integrating new PROMs into routine practice to obtain such data requires significant effort and expense, and the justification can be bolstered by exploration of change over time in response to known events or interventions.

The COVID-19 pandemic provides a natural experiment to explore the responsiveness of the PHE to pandemic-related healthcare disruptions. The COVID-19 pandemic had a complex and substantial impact on health and healthcare behaviors. Public health messaging to slow the spread of COVID-19 increased the salience of health promotion behaviors such as handwashing, social distancing, and masking. The efforts to understand and enact these behaviors represents an increase in engagement behaviors. Simultaneously, there were substantial disruptions to healthcare services and high rates of delayed care or foregone care [2, 22, 23]. Because healthcare engagement is a context-dependent construct that is influenced by both individual capabilities as well as the demands of the healthcare system, it follows that the PHE should capture pandemic-related changes to healthcare services.

The aim of the present study is a preliminary investigation of the PHE’s responsiveness among a heterogeneous group of VA primary care users with mental health and chronic conditions who completed the PHE before and during the COVID pandemic. There is no gold-standard measure of healthcare engagement, so we use the construct approach [19, 20] and compare known groups on their changes in PHE based on their level of COVID-related healthcare disruption. We hypothesized that groups who reported no health care disruption would show increased healthcare engagement, while those with more healthcare disruptions would show proportional decreases in healthcare engagement, with the greatest decreases among those have reported multiple (3 or more) disruptions. These effects were assumed to be conditional on other factors that impact healthcare engagement and that were associated with health-related changes during the COVID pandemic, including changes to physical and mental health during COVID, and sociodemographic factors.

## Methods

### Participants and procedure

These data were part of a larger study of healthcare engagement [18]. Baseline data were collected via a national mail survey between October 2018 through January 2019, to Veterans receiving VA care for mental health (depression, posttraumatic stress disorder) or chronic conditions (diabetes, hypertension). Follow-up data were collected via an online survey during the latter half of the COVID-19 public health emergency, from April 2021 through July 2022. During this period, cases and hospitalizations had been steadily dropping since the second wave of the pandemic [6, 7] though national US household surveys still indicated substantial reports of COVID-related healthcare disruption [6]. The expansion of VA telehealth services had stabilized (May 2021) [11] and vaccines were widely released. This period also included the Delta (July 2021) and Omicron (November 2021) surges.

In the present study, we randomly selected 6640 Veterans who had previously participated in the baseline mail survey, inviting them to participate in a follow-up online survey. Of the 6640 recruitment postcards mailed, 885 Veterans completed the follow-up online survey. Resampling participants allows us to discriminate reliable changes in PHE T-scores over the period between baseline and follow up. Inclusion criteria included participation in the baseline mail survey and a valid mailing address in VA administrative data. Veterans who did not have access to the internet were unable to participate in the follow-up online survey. We began recruitment by mailing 1000 postcards, followed by subsequent waves of postcard mailings monthly. Approximately one week after the postcards were mailed, Veterans were sent an email reminder. On the survey website, Veterans were first presented with an online information sheet that provided all the information that would be included in a standard consent form. Veterans who indicated their willingness to participate in the study completed the online survey. Upon completing the survey, respondents were emailed a $10 electronic Amazon gift card as compensation for their time. This study was approved by the Stanford University School of Medicine Institutional Review Board.

### Data sources and measures

#### PROMIS healthcare engagement

The PROMIS Healthcare Engagement (PHE) short form [18] is an eight-item measure, administered at both time points^1^. Items address engagement behaviors, e.g., “When I need more information I ask, even when my provider is in a rush” or “I make sure I understand all of my test results.” Each item is rated on a 4-point Likert-type scale (*not at all true*, *a little bit true*, *somewhat true*, *mostly true*, and *very true*) with IRT-based scoring yielding standardized T-scores ranging from 0 to 100 (M = 50, SD = 10). The PHE demonstrates good reliability and test-retest stability (ICC = 0.89). Higher scores on the PHE have demonstrated prospective associations with fewer primary care no-shows, better medication adherence, and better chronic condition management [18].

#### COVID-related healthcare disruption

COVID-related disruptions to healthcare was assessed using questions adapted from a public poll of emergency care concerns amidst COVID-19 [1] and from the second phase of the 2020 COVID-19 Household Pulse Survey [8] during the follow-up survey (Time 2). Three questions assessed whether or not respondents encountered a problem in the past three months for each item (i.e., trouble getting an appointment, trouble filling a prescription, and having to get care via phone or video instead of in-person because of COVID). Response options include *a problem*, *not a problem*, or *did not need this type of care*. Two additional questions asked respondents if in the past four weeks, they delayed care or did not receive needed health care due to COVID-19. Responses were summed to reflect disruptions to care: 0 disruptions, 1 disruption, 2 disruptions, 3 or more disruptions.

#### Health status

Physical health was assessed using the PROMIS Global Health – Physical Health Short Form 2a. The two-item measure assesses patient ratings of their overall physical health and how well they can perform physical activities [15]. Mental health was examined using the PROMIS Global Health – Mental Health Short Form 2a, a two-item instrument that examines patient ratings of their overall mental health as well as their satisfaction with social activities and relationships [15]. The PROMIS physical health and mental health measures were administered at both timepoints. T-scores were obtained via the Health Measures scoring service.

#### Health literacy

Health literacy was assessed through a one-item screener at baseline (Time 1): “How confident are you filling out medical forms by yourself” [9]. Responses were scored on a 5-point Likert scale from 1 (*not at all*) to 5 (*extremely*). The lower three response options were grouped to indicate limited health literacy [9].

#### Demographics and COVID-related factors

Demographic characteristics were self-reported from the baseline survey (Time 1) and supplemented with VA administrative data for each Veteran. COVID-related variables (e.g., vaccination status, prior infections) were self-reported from the follow-up survey (Time 2).

### Data analysis

We modeled change in PHE T-scores from baseline to follow-up as a function of the number of COVID-related healthcare disruptions and covariates using mixed effects linear models. These models are more appropriate methods to examine change in observational studies relative to comparing change scores across groups [26]. Because PHE scores are standardized T-scores, regression coefficients represent average marginal effects, which can be interpreted as covariate-adjusted change scores. We calculated marginal (least squares) means, standard errors, and 95% confidence intervals to compare groups on change in PHE scores. Seven Veterans reported that they did not want or need care at follow-up and were therefore missing data for COVID-related disruption and excluded from these analyses. Change in PHE scores did not cluster by VA facility or US region, so analyses did not account for clustering on these variables. All analyses were survey adjusted to account for the stratified sampling.

## Results

### Participants

A total of 885 Veterans completed surveys during the COVID-19 period. Their demographic characteristics are displayed in Table 1. The sample generally reflected the demographic characteristics of the larger baseline sample, where the majority of participants were White (64%), male (72%), and completed some college or technical school (39%). At baseline, physical and mental health functioning was approximately − 1.5 SD below the mean for the US population, consistent with other samples of health care users receiving care for mental health and chronic conditions [16]. At baseline, the mean engagement T-score was 50.66 (raw score = 24.96) with a standard deviation of 9.47. The mean engagement T-score at follow-up was 52.13 (raw score = 25.90) with a standard deviation similar to baseline of 9.81. From baseline to follow-up, the mean change in T-scores was 1.46 (SD = 9.03, range: -35.41 to31.16).

**Table 1:** Sample demographics and descriptive statistics

Variable	*n* (%)
Gender	
Male	635 (71.91)
Female	248 (28.09)
Age group, years	
< 44	129 (14.61)
45–64	289 (32.73)
65–74	260 (29.45)
75+	205 (23.22)
Race/Ethnicity	
Hispanic	124 (14.04)
Black	189 (21.40)
White	562 (63.65)
Other	8 (0.91)
Education	
High school/GED	119 (13.51)
Some college or technical school	345 (39.16)
College graduate or higher	417 (47.33)
Financial strain	
Comfortable	286 (32.57)
Income can provide for basic needs	406 (46.24)
Difficult to get by on present income	129 (14.69)
Very difficult to get by on present income	57 (6.49)
Rurality	
Urban	605 (68.52)
Rural	278 (31.48)
Health literacy	
Adequate	660 (76.39)
Limited	204 (23.61)
PROMIS Global Physical, mean (SD)	43.67 (8.23)
PROMIS Global Mental, mean (SD)	43.45 (10.07)
Chronic conditions	
0	84 (9.51)
1	128 (14.50)
2	157 (17.78)
3	133 (15.06)
4	135 (15.29)
5+	246 (27.86)
**COVID-related factors**	
Disruptions to care^a^	
0	509 (57.64)
1	197 (22.31)
2	98 (11.10)
3+	79 (8.95)
Vaccination intention^a^	
Yes/Already received	759 (86.84)
No	80 (9.15)
Don’t know	35 (4.00)
Spoke with doctor about vaccine^a^	
Yes	510 (58.22)
No	357 (40.75)
Don’t know	9 (1.03)
Past two weeks smartphone/computer video with:^a^	
Friends or family	581 (65.80)
Co-worker(s)	152 (17.21)
Doctor/healthcare provider	399 (45.19)
Contracted COVID-19^a^	
Yes	29 (3.42)
No	818 (96.58)

*Note* All variables are Time 1 (baseline) unless noted otherwise

^a^Variables measured at Time 2 (follow-up)

### Changes in healthcare engagement

On average, population healthcare engagement scores increased modestly by 2.84 points (*SE* = 0.39; *t* = 7.26, *p* < 0.001). Table 2 displays the regression coefficients representing adjusted change scores at each level of healthcare disruption. Each group significantly differed from the 0 disruptions group. Comparisons among groups of individuals who experienced healthcare disruption revealed that groups with greater numbers of disruptions showed significantly greater decreases in engagement scores. Those who reported 2 disruptions showed greater decreases relative to those reporting only 1 disruption (difference = -4.20, *SE* = 0.92; *t* = 4.58, *p* < 0.001) and those who reported 3 or more disruptions showed greater decreases in engagement compared to those that reported 2 disruptions (difference = -3.75, *SE* = 1.23; *t* = 3.04, *p* = 0.002). Increases in healthcare engagement were slightly but significantly greater among Hispanic Veterans and older Veterans, and somewhat lower among Veterans with limited health literacy. See Supplemental Table 1 for full regression output.

**Table 2:** Group differences from individuals without COVID-related healthcare disruptions

Healthcare disruptions	Change score	SE	t	*p*-value
0 (reference)	-	-	-	-
1	-1.98	0.66	-3.01	0.003
2	-5.36	0.82	-6.49	< 0.001
3+	-8.11	1.08	-7.48	< 0.001

*Note* Change scores reflect regression coefficients; scores are adjusted for age, race/ethnicity, health literacy, rurality, and changes in physical and mental health functioning. *SE* standard error, *CI* confidence interval

## Discussion

We evaluated the PHE’s responsiveness through comparing changes in healthcare engagement during the COVID-19 pandemic between Veterans reporting healthcare disruptions and Veterans who did not report healthcare disruptions. Our results show that Veterans with mental health and chronic conditions using VA care demonstrated pronounced shifts in healthcare engagement. Our hypothesis that Veterans reporting COVID-related healthcare disruptions, such as difficulties scheduling appointments or delaying care, would demonstrate decreases to healthcare engagement was also supported. Our results also revealed that such disruptions had a dose-response effect, such that groups who reported more disruptions also demonstrated greater, negative changes to healthcare engagement. These results are consistent with prior research that has shown that there were substantial changes to healthcare service delivery during the pandemic [2, 11, 23]. We also observed that healthcare engagement scores increased during the pandemic among Veterans who did not report COVID-related healthcare disruptions. This finding is reflective of the healthcare experiences during the latter half of the pandemic. Improved access to COVID-related resources (e.g., vaccines, telehealth) [11], along with greater adoption of individual health promotion behaviors [14], likely contributed to increased healthcare engagement. Our results indicate that the PHE is sensitive to the effects of healthcare service delivery changes on healthcare engagement, providing preliminary evidence for the PHE’s responsiveness.

Our results revealed changes in healthcare engagement from pre- to peri-COVID, providing evidence for the PHE’s responsiveness to environmental influences such as healthcare disruptions. The ability to accurately assess shifts in healthcare engagement has potential to aid providers and healthcare systems in tailoring care and tracking the effectiveness of interventions. The PHE’s responsiveness to environmental changes adds to several other previously established strengths of the PHE for assessing healthcare engagement. The PHE uses broad, general language that can be administered in different hospital and clinical contexts; is relatively short (eight items); and can be administered in person or virtually [18]. Importantly, the PHE was constructed at a sixth-grade reading level to preserve accessibility to low literacy patients. Our results indicate that Veterans with limited health literacy demonstrated slightly lower changes in healthcare engagement from pre- to peri-pandemic, consistent with existing research finding that low literacy is associated lower likelihood of adopting preventive health behaviors [21]. Accurate assessment of healthcare engagement among low literacy patients is essential to providing equitable care, and the PHE can be used to assess healthcare engagement among patients with limited health literacy.

The national scope of our study is a strength, but our findings may not generalize beyond VA users. Notably, VA healthcare provides healthcare coverage and access to a large nationwide integrated healthcare system, which means our sample may have been less affected by the pronounced economic inequalities that characterized the COVID pandemic. VA was among the first healthcare providers to receive vaccines, and was able to coordinate an early rollout with a focus toward engaging high-risk and underserved Veterans to minimize disparities [25]. VA had also been building a telehealth program to engage rural Veterans since the mid-2000s [10], which may have facilitated a smoother transition to telehealth services during the pandemic.

Results should also be interpreted in light of the natural experiment study design. The unexpected and pervasive nature of the COVID pandemic made controlled studies of its impact impossible. We were fortunate to be able to capitalize on a baseline assessment prior to COVID to assess the impact of the pandemic on healthcare engagement. These findings expand upon prior work that attests to the validity and test-retest stability of PHE scores [18], suggesting the observed changes in PHE scores reflect pandemic influences. Arguably, had we not detected changes in PHE scores during such a global healthcare state of emergency, we would conclude that the PHE had limited utility. Because responsiveness, like other aspects of construct validity, is an ongoing process [19], future research should build on the present study’s results to further investigate the PHE’s responsiveness. Nonetheless, our results provide preliminary support for the PHE’s responsiveness to substantial influences on health services.

## Conclusion

Healthcare engagement reflects patient self-efficacy for enacting behaviors to obtain optimal benefit from health services. The PHE is a new measure of healthcare engagement that has previously shown excellent content and construct validity. The present study adds to the PHE’s utility as a measure of healthcare engagement by demonstrating that the PHE is responsive to changes in health services. These results support the PHE as a measure that can be used to assess patient engagement and track engagement over time. Though future research should investigate whether patient engagement can be modified by intervention, the results of the present study suggest that the PHE is responsive to healthcare changes and can be used to inform treatment decision-making and maximize quality of care.

## Electronic supplementary material

Below is the link to the electronic supplementary material.

Supplementary Material 1

## Data Availability

Data will be made available in accordance with the data management plan submitted to the VA Office of Research & Development. Within one year of publication of manuscripts addressing the aims of the grant, investigators will make a deidentified, anonymized dataset available to the public.

## Abbreviations

CDW: Corporate Database Warehouse
PROMIS: Patient Reported Outcomes Measurement Information System
PHE: PROMIS Healthcare Engagement
VA: Veteran’s Health Administration

## Footnotes

1 The PHE is not yet accessible on the Health Measures platform but is available from the last author (rachel.kimerling@va.gov) upon request.
