# Supplementary material for: Changes in healthcare engagement during the COVID-19 pandemic
Source: J Patient Rep Outcomes. 2025 Feb 20;9:21. doi: 10.1186/s41687-025-00850-z (PMC11842638; doi:10.1186/s41687-025-00850-z)
Supplement: Supplementary file 1 — Supplementary Material 1 [file 41687_2025_850_MOESM1_ESM.docx]

**Supplemental Table**

**Table.** Changes in PROMIS Healthcare Engagement (PHE) scores from mixed model

| **Variable** | **Coefficient** | ***SE*** | ***t*** | **95% CI** | ***p*** |
| --- | --- | --- | --- | --- | --- |
| Time | 2.83 | 0.39 | 7.26 | 2.07 to 3.60 | < 0.001 |
| COVID-related healthcare disruptions | | | | | |
| 0 (reference group) | - | - | - | - | - |
| 1 | -1.98 | 0.66 | -3.01 | -3.26 to -0.68 | 0.003 |
| 2 | -5.36 | 0.82 | -6.49 | -6.98 to -3.74 | 0.000 |
| 3+ | -8.10 | 1.08 | -7.48 | -10.23 to -5.98 | 0.000 |
| Health literacy | -2.49 | 0.59 | -4.22 | -3.67 to -1.33 | 0.000 |
| Race/Ethnicity | | | | | |
| White (reference group) | - | - | - | - | - |
| Hispanic | 1.52 | 0.78 | 1.94 | -0.02 to 3.06 | 0.05 |
| Black | 0.59 | 0.65 | 0.90 | -0.69 to 1.86 | 0.37 |
| Age group, years | | | | | |
| < 44 | - | - | - | - | - |
| 45–64 | 1.22 | 0.80 | 1.53 | -0.35 to 2.79 | 0.13 |
| 65–74 | 3.46 | 0.80 | 4.38 | 1.91 to 5.01 | 0.000 |
| 75+ | 2.57 | 0.82 | 3.15 | 0.97 to 4.18 | 0.002 |
| Rurality | 0.08 | 0.53 | 0.14 | -0.97 to 1.12 | 0.89 |
| PROMIS Global Mental | 1.94 | 0.23 | 8.43 | 1.49 to 2.39 | 0.000 |
| PROMIS Global Physical | 1.75 | 0.25 | 7.14 | 1.26 to 2.23 | 0.000 |

*SE* standard error, *CI* confidence interval
